# Supplementary material for: Synaptic density marker SV2A is reduced in schizophrenia patients and unaffected by antipsychotics in rats
Source: Nat Commun. 2020 Jan 14;11:246. doi: 10.1038/s41467-019-14122-0 (PMC6959348; doi:10.1038/s41467-019-14122-0)
Supplement: Supplementary file 1 — Supplementary Information [file 41467_2019_14122_MOESM1_ESM.pdf]

Supplementary Information

Synaptic density marker SV2A is reduced in schizophrenia patients and unaffected by antipsychotics in rats

Onwordi et al.

Supplementary Figures

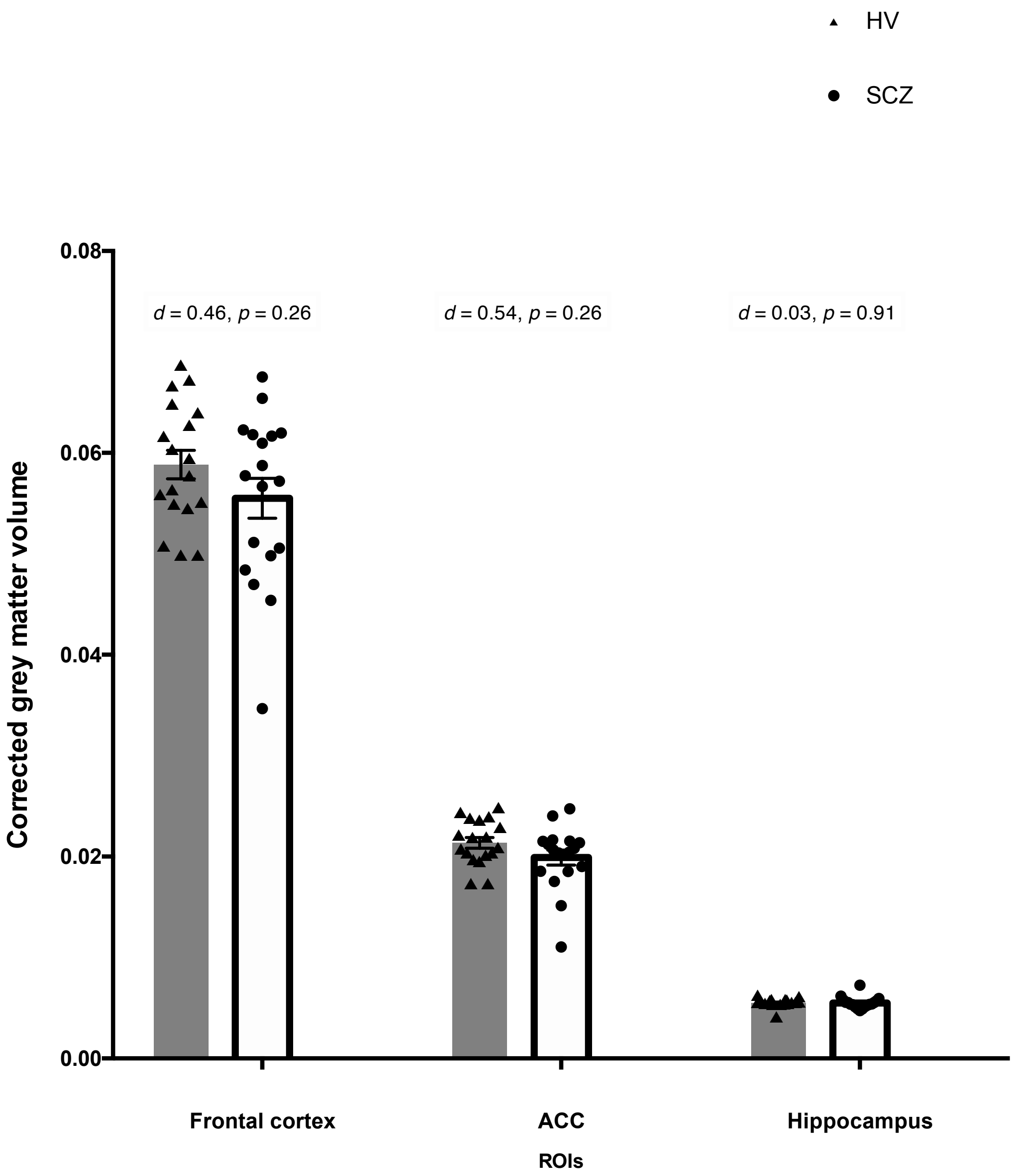

Supplementary Figure 1 – Regional mean corrected grey matter volume by group. Grey bars depict regional mean corrected grey matter volume in the healthy volunteer (HV) group, and triangles represent individual HV values (n = 18). Hollow bars depict mean corrected grey matter volume in the schizophrenia (SCZ) group, and circles indicate individual SCZ patient values (n = 18). FDR-adjusted p values from post hoc t-tests are reported here. Corrected grey matter volume was not significantly altered in the SCZ compared to the HV group in the frontal cortex, anterior cingulate cortex (ACC) or hippocampus ($p > 0.05$). Error bars indicate standard error of the mean.

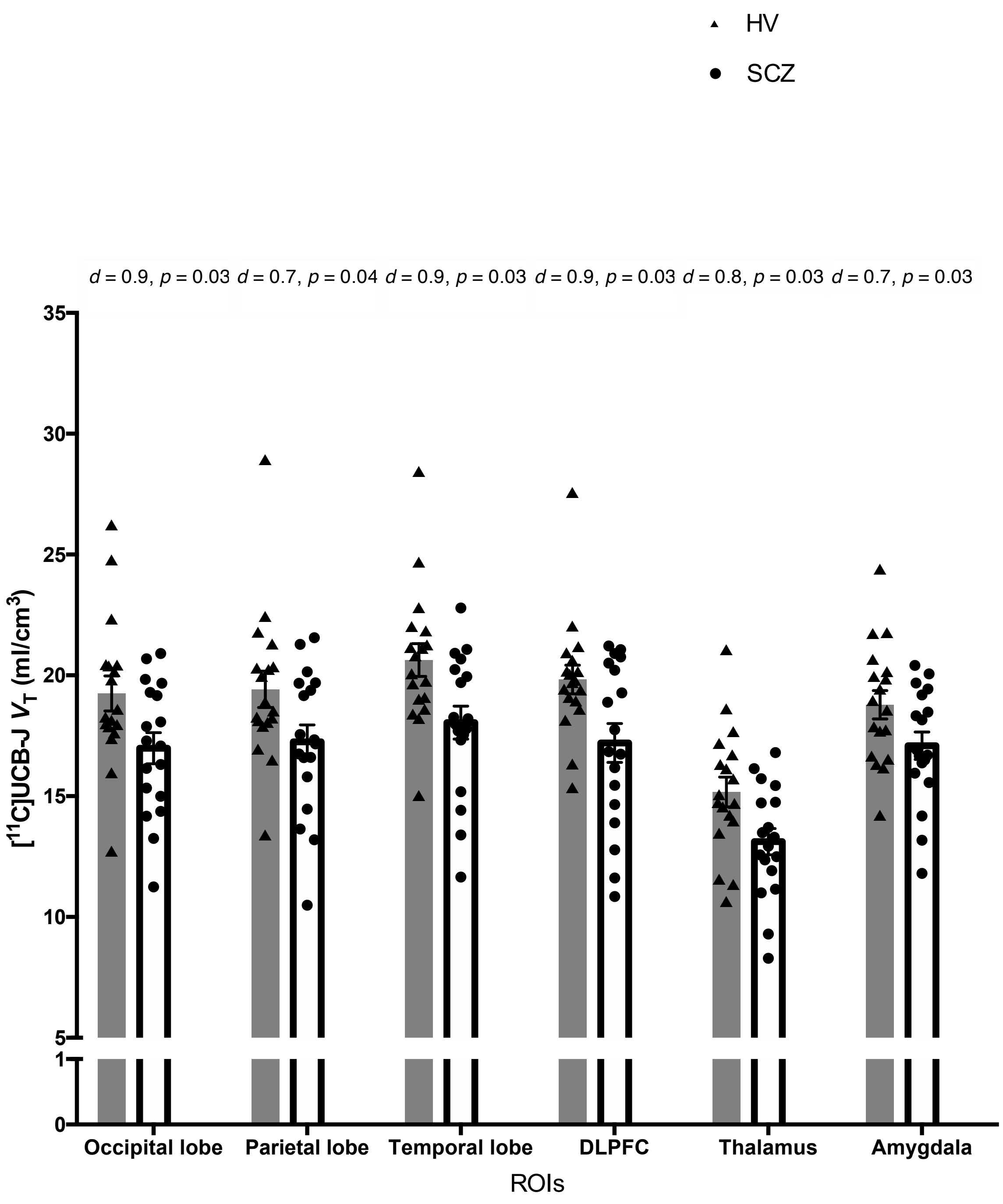

Supplementary Figure 2 – [^{11}C]UCB-J distribution volume (V_T) in exploratory regions of interest (ROIs). The occipital, parietal and temporal lobes, dorsolateral prefrontal cortex (DLPFC), thalamus and amygdala were assessed for group differences. Grey bars depict regional mean V_T in the healthy volunteer (HV) group, and triangles represent individual HV V_T (n = 18). Hollow bars depict mean V_T in the schizophrenia (SCZ) group, and circles indicate individual SCZ patient V_T (n = 18). FDR-adjusted p values from post hoc t-tests are reported here. [^{11}C]UCB-J V_T was significantly reduced with moderate-to-large effect sizes in the SCZ compared to the HV group in each of the regions of interest (ROIs). Error bars indicate standard error of the mean.

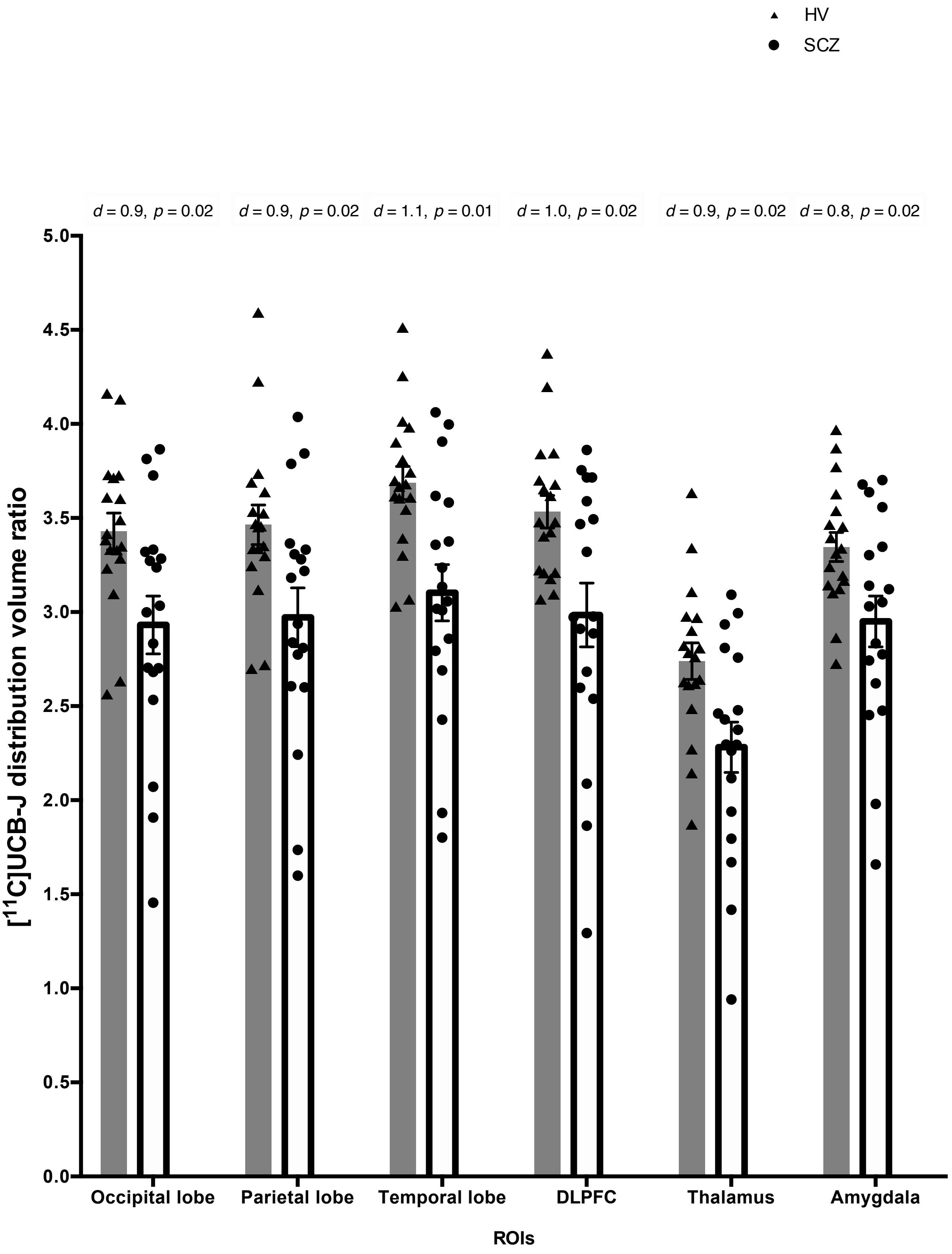

Supplementary Figure 3 – [11C]UCB-J distribution volume ratio (DVR in exploratory regions of interest (ROIs). The occipital, parietal and temporal lobes, dorsolateral prefrontal cortex (DLPFC), thalamus and amygdala were assessed for group differences. Grey bars depict regional mean DVR in the healthy volunteer (HV) group, and triangles represent individual HV DVR (*n* = 18). Hollow bars depict mean DVR in the schizophrenia (SCZ) group, and circles indicate individual SCZ patient DVR (*n* = 18). P values reported here are FDR-adjusted *p* values. [11C]UCB-J V_T was significantly reduced with large effect sizes (Cohen's *d* > 0.8) in the SCZ compared to the HV group in each of the ROIs. Error bars indicate standard error of the mean.

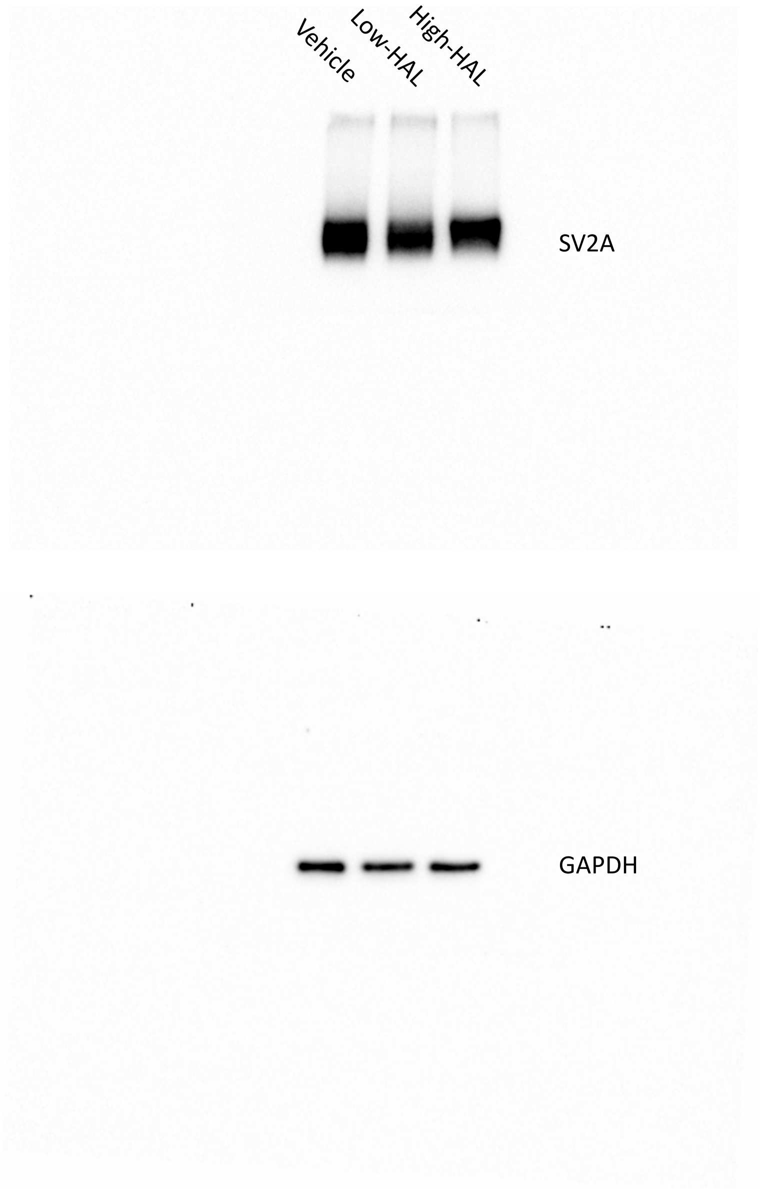

Supplementary Figure 4 – unprocessed western blot images of SV2A and GAPDH protein levels in synaptosome fractions purified from the rat frontal cortex. Related to Figure 4A.

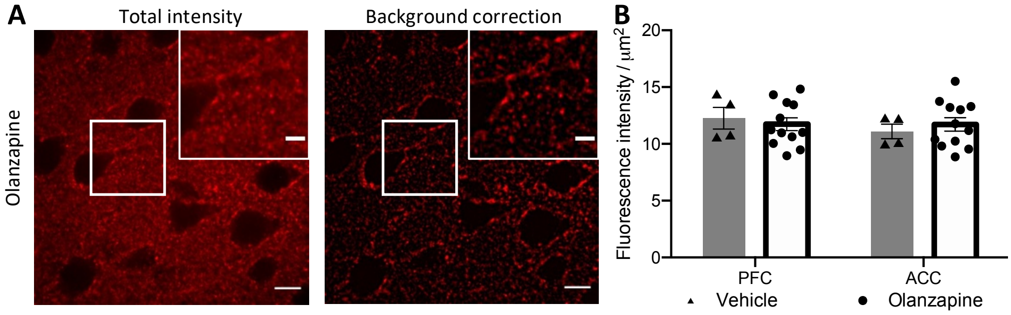

Supplementary Figure 5 – Chronic olanzapine administration does not affect SV2A levels in the rat frontal cortex. A. Confocal images of brain sections from olanzapine-treated animals, showing immunostaining for SV2A (red). Both the original image (left hand side) and background corrected image (right hand side) are shown. Scale bar, 10 µm (large field of view) or 4 µm (zoomed insets). **B.** Quantification of background corrected SV2A intensity values. There was no significant effect of olanzapine treatment on SV2A immunostaining intensity (two-way ANOVA, effect of treatment: $F_{1,14} = 0.0018$, $p = 0.97$). Error bars indicate standard error of the mean.

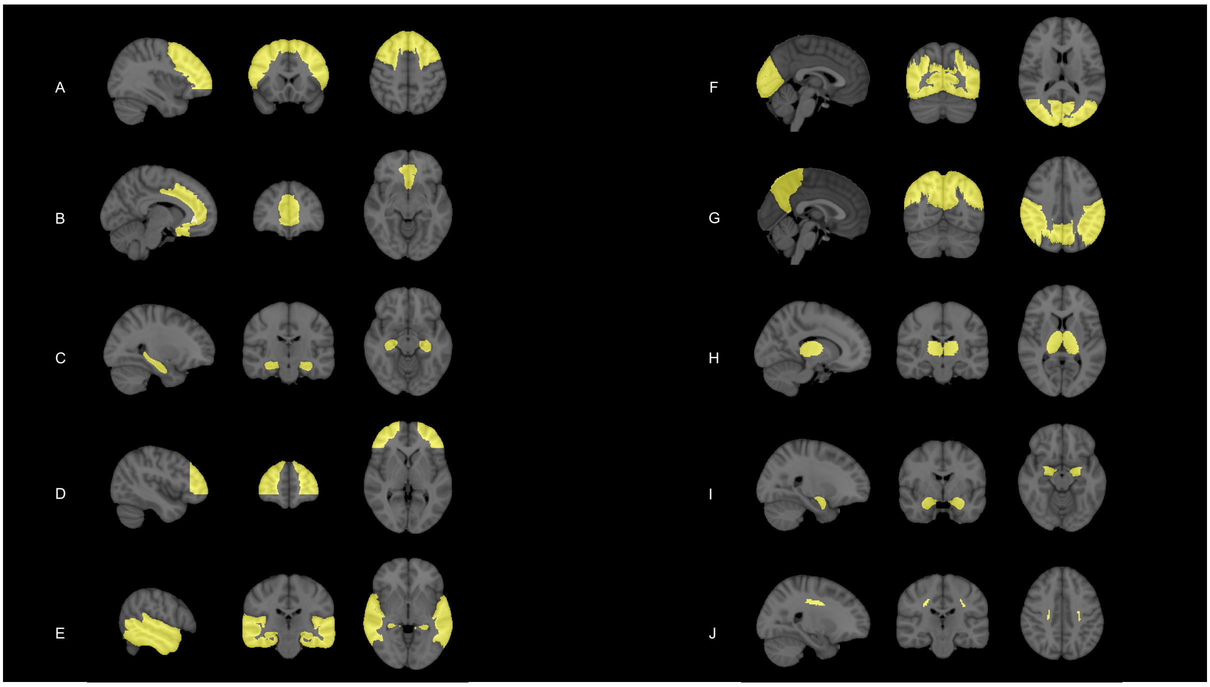

Supplementary Figure 6 – Regions of interest (ROIs) assessed using [^{11}C]UCB-J PET. Images of the ROIs from the Clinical Imaging Centre (CIC) atlas (A-I) and automated anatomical labelling template (J) are highlighted in yellow and superimposed on the associated MNI152 template MRI sagittal, coronal and transverse images. A – frontal cortex; B – anterior cingulate cortex; C – hippocampus; D – dorsolateral prefrontal cortex; E. – temporal lobe; F – occipital lobe; G – parietal lobe; H – thalamus; I – amygdala; J – centrum semiovale.

Supplementary Table

Subjects (SCZ)	Current antipsychotic	Daily dose (mg)	DDD-derived chlorpromazine equivalent dose (mg)[1]
1	Clozapine	1400	1400
2	Risperidone	3.6	213.9
3	Clozapine	400	400
4	Olanzapine	10	300.3
5	Clozapine	350	350
6	Olanzapine	20	600.6
7	Aripiprazole	10	200
8	Paliperidone	11.7	1405.7
9	Clozapine	500	500
10	Risperidone	3.6	213.9
11	Depixol	5.7	285.7
12	Clozapine	550	550
13	Paliperidone	3.6	178.6
14	Lurasidone	37	125
15	Paliperidone	3.2	160.7
16	Olanzapine	20	600.6
17	Paliperidone	1.6	198.1
18	Clozapine	300	300
Mean (SEM) dose			467.8 (99.3)

Supplementary Table 1 – current antipsychotic drug treatment, daily dose and DDD-derived chlorpromazine equivalent dose.

Supplementary methods:

Animals and antipsychotic drug administration

Animal experiments were carried out in accordance with the Home Office Animals (Scientific Procedures) Act (1986) and European Union (EU) Directive 2010/63/EU, with the approval of the local Animal Welfare and Ethical Review Body (AWERB) panel at King's College London (KCL).

Male Sprague-Dawley rats (Charles River UK Ltd, Margate, UK), initial body weight 240-270 g (6 to 10 weeks of age), were housed four per cage in ventilated plastic cages (38 x 59 x 24 cm, Tecniplast, UK) containing sawdust, paper sizzle nest and cardboard tunnels (Datesand group, UK). Animals were maintained under a 12-hour light/dark cycle (0700 lights on) with food and water available ad libitum. Room temperature and humidity were maintained at 21 ± 2°C and 55 ± 5%, respectively. Animals were habituated for 7 days before experimental procedures.

Experimental Design (animals)

A cross-sectional design was employed in which vehicle (β-hydroxypropylcyclodextrin, 20% wt/vol, acidified by ascorbic acid to pH 6), haloperidol (0.5 and 2 mg per kg per day; Sigma-Aldrich, Gillingham, UK) or olanzapine (7.5 mg per kg per day; Sigma-Aldrich, Gillingham, UK) were administered using osmotic minipumps for 28 days (equivalent to approximately 2.5 human years based on 11.8 rat days as equivalent to 1 human year [2]). The doses of haloperidol and olanzapine were chosen based on previous dopamine D2 receptor (D2R) occupancy studies [3]; and serum plasma levels achieved following chronic administration using these doses reflect D2R occupancy in the range 70-90%, in line with typical D2 occupancy used in clinical treatment [3-5]. The osmotic pump delivers drug or vehicle at a steady rate in comparison to once-daily injections where drug-levels fall to undetectable levels within 24 hours, based on a half-life of 2.5 hours for most antipsychotic drugs in rats [3]. Minipumps (Alzet Model 2ML4; 28 days; Alzet, Cupertino, CA, USA) filled with drug or vehicle solutions were inserted subcutaneously into the back flank under isoflurane anaesthesia (5% induction, 1.5% maintenance delivered in a 70/30% medical air/oxygen mix) [4,5]. After 28 days' exposure, animals were terminally anaesthetised by injection of sodium pentobarbital (60 mg per kg, intraperitoneal) and culled by cardiac perfusion using

heparinised ice-cold 0.9% saline [4,5]. A blood sample was collected at termination for estimation of drug levels, measured using tandem mass spectrometry (Cyprotex, Macclesfield, UK). Three cohorts of vehicle and drug-exposed rats were generated for different experimental end-points. Specifically, cohort 1 comprised vehicle- (*n* = 10); 0.5 mg per kg per day (*n* = 12) or 2 mg per kg per day haloperidol- (HAL) (*n* = 11) treated groups, with brain tissue utilised for western blotting and, in a subset of these animals, for [^{3}H]UCB-J autoradiography (vehicle, *n* = 7; 0.5 mg per kg per day HAL, *n* = 3; 2 mg per kg per day HAL, *n* = 5). Cohort 2 comprised vehicle- (*n* = 11) and 0.5 mg per kg per day HAL- (*n* = 11) exposed rats, with brain tissue processed for *post-mortem* analysis of SV2A intensity using immunofluorescence staining and confocal microscopy. Cohort 3 comprised vehicle- (*n* = 4) and 7.5 mg per kg per day olanzapine- (*n* = 12) exposed rats. Brain tissue was fixed and processed for immunostaining in the same way as brain tissue from cohort 2.

Post-mortem tissue handling

Following perfusion, brains were extracted and hemisected. For cohort 1, brains were snap-frozen in isopentane (–25 to −30°C) on dry ice and stored at –70°C until further processing for either synaptosome extraction or autoradiography. Brains extracted from cohorts 2 and 3 were drop-fixed overnight in 4% PFA at 4°C. After 18-20h fixation brains were washed once in 0.01M phosphate buffered saline (PBS), and transferred to buffered 30% sucrose solution for 48h at 4°C. Brain hemispheres were then snap-frozen on dry ice and stored at –70°C until further processing for immunostaining.

Synaptosome extraction and western blotting

Synaptosomes were prepared from fresh-frozen brain tissue from each animal in cohort 1. Specifically, the frontal cortices (FC – prefrontal and cingulate cortex) were dissected from fresh-frozen rat brain hemispheres on ice and homogenised in lysis buffer (150 mM NaCl, 1 mM EDTA, 1 mM EGTA, 20 mM Tris pH7.4, 1% Triton-X100, supplemented with a cocktail of protease and phosphatase inhibitors (Pierce, UK)) using a plastic pestle. The resultant homogenates (total lysate) were then centrifuged for 8 min at 2,000 rpm at 4°C to remove nuclei. The resultant supernatants (S1) were then transferred to new tubes and centrifuged for 15 min at 13,000 rpm at 4°C. The resultant pellet (P2) containing crude

synaptosomes was resuspended in 100 µl lysis buffer and protein concentration quantified using a Pierce BCA protein assay kit (Thermo Scientific, UK). 30 µg of protein from each sample was transferred into a fresh Eppendorf containing an equal amount of Laemmli sample buffer (Bio-Rad Laboratories, UK). Samples were denatured at 95°C for 5 minutes on a heat block and separated on a 10% acrylamide gel before transfer onto an Immun-Blot® PVDF membrane (Bio-Rad Laboratories, UK) via wet transfer for 16.5 hours overnight at 4°C. Membranes were blocked for non-specific binding using 5% bovine serum albumin (BSA) in tris-buffered saline containing 0.1% triton (TBS-T). Primary antibodies (rabbit polyclonal α-SV2A, ab32942, Abcam, Cambridge, UK; 1:2000; mouse monoclonal α-GAPDH, 60004-1, Protein Tech, 1:10,000) were left to hybridise overnight at 4°C with agitation. Secondary antibodies (goat-α-mouse, and goat-anti rabbit, both HRP-conjugated, 31430 and 31460 respectively, Pierce, UK, 1:10,000) were incubated at room temperature (RT) for 1 hour diluted in TBS-T containing 5% BSA. To visualise the proteins of interest, membranes were incubated with Clarity Western ECL substrate (Bio-Rad Laboratories, UK) for 5 minutes prior to imaging. Proteins bound to the membrane were visualised using the Biorad ChemiDoc scanner using Image LabTM Software (PC Version 6.0 SOFT-LIT-170-9690-ILSPC). Grey scale images of scanned membranes were processed and quantified using Image Studio (Version 5.2.5, Li-Cor Biosciences). Briefly, a uniform sized box was traced around each band detected by the scanner to provide an intensity value. Background intensity was then subtracted by taking the median of background pixel intensities in the selected background regions of the bands. SV2A protein intensity was then normalised to that of GAPDH as the loading control for each rat in each group. See Supplementary Figure 4 for unprocessed western blot images of SV2A and GAPDH protein levels in synaptosome fractions purified from the rat frontal cortex.

Ex-vivo autoradiography for SV2A using [^{3}H]UCB-J

From the opposite intact brain hemispheres of a subset of the same animals in cohort 1, coronal sections (20 µm-thick) were cut on a cryostat and thaw mounted on 1% gelatinized superfrost slides. Individual slides containing 10-12 adjacent tissue sections at 500 µm interval were then preincubated in assay buffer (50mM Tris Base, 140mM NaCl, 1.5mM MgCl2, 5mM KCl, 1.5mM CaCl2, pH 7.4) for 10 min at RT, and subsequently incubated for

2 hr at RT in assay buffer containing either [^{3}H]UCB-J (12.5nM), or [^{3}H]UCB-J in the presence of levetiracetam (Sigma, Gillingham, UK; diluted to 1 mM in DMSO) to assess total and non-specific binding, respectively. After incubation, slides were washed twice in ice-cold wash buffer (50mM Tris Base, 1.4mM MgCl2, pH 7.4), followed by a brief rinse in ice-cold reverse osmosis (RO) water and dried in a cool airstream. Sections were apposed to Carestream Kodak Biomax Light film (Sigma-Aldrich) adjacent to tritium microscale standards (American Radiolabeled Chemicals) for 6 weeks before the autoradiograms were developed and analysed using MCID Basic 7.0 software (Interfocus, Cambridge, UK). A standard curve was derived from densitometry of the adjacent tritiated standards corrected for the specific activity of the radioligand. Specific binding (fmol per mg tissue) was calculated by subtracting the non-specific from total binding. For each brain, specific binding of SV2A was measured in two pre-specified regions of interest (ROI), the prefrontal cortex and anterior cingulate cortex (selected because these were the ROIs selected *a priori* in which significant differences in V_T were seen in schizophrenia in our clinical study) across three consecutive coronal sections and the mean specific binding calculated.

Fluorescence immunostaining

The intact left hemisphere of each animal from cohort 2 and 3 was serially sectioned (30 µm-thick, interval 1/12, 360 µm spacing between sections) on a cryostat at -20°C and stored in tissue cryoprotection solution (25% glycerol, 30% ethylene glycol, 45% 1x PBS pH 7.4, 0.05% azide) at -20°C until further processing. Free-floating sections from each brain in each group were washed for 10 min in phosphate buffer (PB; 0.1M) and 2x10 min in 1x PBS. For antigen retrieval sections were incubated for 10-15 mins in 10 mM sodium citrate (pH 6.2) at RT, followed by incubation in pre-heated 10 mM sodium citrate (pH 6.2) in a water-bath at 78°C. Sections were then allowed to cool down to RT in the same solution while gently shaking for 30 min. Sections were then washed twice in PBS (2 x 5') supplemented with 0.05% Triton-X100 and incubated for 4 hr in blocking solution (10% NGS, 1.5% BSA, 0.3% Triton-X100 in PBS). Sections were then incubated for 18h at 4°C with primary antibody (Rabbit-α-SV2A, Abcam ab32942; 1:1000) diluted in blocking solution supplemented with 0.02% sodium azide. Specificity of the SV2A antibody in immunostaining has previously been verified using slices from SV2A KO mice, as well as SV2A blocking peptides[6].

Sections were then washed in PBS (3 x 10') and incubated for 2h in secondary antibody solution. Sections were then mounted on Superfrost Plus slides (ThermoFisher) and air-dried at RT for 1 hr before coverslipping with mounting medium containing DAPI (Vectashield).

Confocal image acquisition and analysis

Images of SV2A staining were acquired using an Inverted Spinning Disk confocal microscope (Nikon, JP) using a 60x oil immersion lens objective (NA 1.4). Images were 102.65 x 102.65 µm in size (512 x 512 pixels), acquired as a stack spanning 6-10 µm, at an interval of 0.3 µm. Image stacks were acquired from 4-5 consecutive sections containing the pre-specified brain regions, either the PFC, (Bregma +4.2 to +2.5mm, 6 stacks per section) or the ACC (Bregma +2.3 to +0.0 mm, 4 stacks per section). SV2A intensity was analysed using an in-house written macro in ImageJ (https://imagej.net/Welcome). Total SV2A staining intensities were measured from 3 consecutive optical sections within each image stack, selected based on quality of staining and contrast in the image, to ensure proper synaptic staining. These optical sections were then combined by maximum intensity projection. Total SV2A intensity was measured after background subtraction using a rolling ball with radius of 25 pixels (5 µm) to remove diffuse (and presumably non-synaptic) staining.

Supplementary Notes:

Supplementary Note 1: effect of smoking on [^{11}C]UCB-J V_T in a priori ROIs

A significantly greater proportion of the schizophrenia group were current smokers as compared to the healthy volunteer group (p = 0.002, two-tailed Fisher's exact test). In view of this we conducted an exploratory analysis of the effect of smoking status on [^{11}C]UCB-J V_T. This showed there was a significant effect of ROI (two-way ANOVA: $F_{1.5, 23.7}$ = 129.9, p < 0.0001), but no significant effect of smoking ($F_{1, 16}$ = 1.50, p = 0.24) nor smoking-by-ROI interaction ($F_{1.5, 23.7}$ = 0.68, p = 0.48) on [^{11}C]UCB-J V_T. Post hoc analyses with false-discovery rate (FDR) adjustment revealed that mean (SEM) [^{11}C]UCB-J V_T (ml/cm^3) was not significantly altered in the smokers relative to the non-smokers in the FC (smokers = 16.19 [1.08]; non-smokers = 18.42 [0.88]; t = 1.34, df = 16.0, p = 0.32 [two-tailed independent samples t-test]), the ACC (smokers = 19.01 [1.08]; non-smokers = 20.64 [0.54]; t = 1.03, df = 16.0, p = 0.32) or hippocampus (smokers = 13.59 [0.74]; non-smokers = 15.08 [0.96]; t = 1.19, df = 16.0, p = 0.32).

Supplementary Note 2: effect of concomitant psychotropic medications on [^{11}C]UCB-J V_T in a priori ROIs

Five of the schizophrenia patients were taking other psychotropic drugs in addition to antipsychotics (Supplementary Data 1). Although none of these drugs are known to bind to SV2A, we conducted an exploratory analysis in case there was an indirect effect of concomitant medication on [^{11}C]UCB-J V_T. This showed there was no significant effect of concomitant psychotropic medication (two-way ANOVA: $F_{1, 16}$ = 1.58, p = 0.23) nor concomitant psychotropic medication-by-ROI interaction ($F_{2, 32}$ = 0.20, p = 0.82) on [^{11}C]UCB-J V_T. Post hoc analyses with FDR-adjustment revealed that mean (SEM) [^{11}C]UCB-J V_T (ml/cm^3) was not significantly altered in those taking concomitant psychotropic medications (CPM) relative those not taking concomitant psychotropic medications (nCPM) the FC (nCPM = 17.50 [0.82]; CPM = 15.46 [1.95]; t = 1.15, df = 16.0, p = 0.27 [two-tailed independent samples t-test]), ACC (nCPM = 20.13 [0.64]; CPM = 18.06 [2.15]; t = 1.26, df = 16.0, p = 0.27) or hippocampus (nCPM = 14.55 [0.63]; CPM = 12.89 [1.35]; t = 1.27, df = 16.0, p = 0.27).

Supplementary Note 3: effect of clozapine treatment on [^{11}C]UCB-J V_T in a priori ROIs

There was a significant effect of ROI (two-way ANOVA: $F_{2, 32} = 126.4$, $p < 0.0001$), but no significant effect of clozapine-treatment ($F_{1, 16} = 1.86$, $p = 0.19$) nor clozapine treatment-by-ROI interaction ($F_{2, 32} = 0.215$, $p = 0.81$) on [^{11}C]UCB-J V_T. Post hoc analyses with false-discovery rate (FDR) adjustment revealed that mean (SEM) [^{11}C]UCB-J V_T (ml/cm^3) was not significantly altered in the non-clozapine treated SCZ relative to the clozapine treated SCZ group in the FC (non-clozapine treated SCZ = 17.67 [0.91]; clozapine-treated SCZ = 15.46 [1.50]; $t = 1.33$, $df = 16.0$, $p = 0.28$ [two-tailed independent samples t-test]), the ACC (non-clozapine treated SCZ = 20.14 [0.86]; clozapine-treated SCZ = 18.38 [1.45]; $t = 1.11$, $df = 16.0$, $p = 0.28$) or hippocampus (non-clozapine treated SCZ = 14.74 [0.70]; clozapine-treated SCZ = 12.79 [0.98]; $t = 1.62$, $df = 16.0$, $p = 0.28$).

Supplementary Note 4: comparison of uncorrected SV2A protein levels between vehicle- and haloperidol-exposed rats as measured by western blots

One-way ANOVA of SV2A intensity on western blot, not corrected for GAPDH signal, revealed no statistically significant differences between the groups ($F_{2,29} = 0.37$; $p = 0.70$).

References

1 Leucht, S., Samara, M., Heres, S. & Davis, J. M. Dose Equivalents for Antipsychotic Drugs: The DDD Method. *Schizophr Bull* **42 Suppl 1**, S90-94, doi:10.1093/schbul/sbv167 (2016).

2 Quinn, R. Comparing rat's to human's age: how old is my rat in people years? *Nutrition* **21**, 775-777, doi:10.1016/j.nut.2005.04.002 (2005).

3 Kapur, S., VanderSpek, S. C., Brownlee, B. A. & Nobrega, J. N. Antipsychotic dosing in preclinical models is often unrepresentative of the clinical condition: a suggested solution based on in vivo occupancy. *J Pharmacol Exp Ther* **305**, 625-631, doi:10.1124/jpet.102.046987 (2003).

4 Vernon, A. C. *et al.* Contrasting effects of haloperidol and lithium on rodent brain structure: a magnetic resonance imaging study with postmortem confirmation. *Biol Psychiatry* **71**, 855-863, doi:10.1016/j.biopsych.2011.12.004 (2012).

5 Vernon, A. C., Natesan, S., Modo, M. & Kapur, S. Effect of chronic antipsychotic treatment on brain structure: a serial magnetic resonance imaging study with ex vivo and postmortem confirmation. *Biol Psychiatry* **69**, 936-944, doi:10.1016/j.biopsych.2010.11.010 (2011).

6 Crevecoeur, J. *et al.* Expression of SV2 isoforms during rodent brain development. *BMC Neurosci* **14**, 87, doi:10.1186/1471-2202-14-87 (2013).
