# Supplementary material for: Synaptic density marker SV2A is reduced in schizophrenia patients and unaffected by antipsychotics in rats
Source: Nat Commun. 2020 Jan 14;11:246. doi: 10.1038/s41467-019-14122-0 (PMC6959348; doi:10.1038/s41467-019-14122-0)
Supplement: Supplementary file 2 — Description of Additional Supplementary Files [file 41467_2019_14122_MOESM2_ESM.docx]

**Description of Additional Supplementary Files**

**Supplementary Data 1:** Individual subject data for schizophrenia (SCZ) and healthy volunteer (HV) subjects on age of symptom onset, duration of illness, current smoking status, concomitant psychotropic medications and symptom severity (PANSS positive, negative and general scores). Asterisks (*) signify psychotropic drugs prescribed for symptoms of anxiety, although these subjects did not achieve SCID criteria for anxiety disorders. Obelisks (†) signify drugs prescribed for anhedonia in subjects who did not achieve SCID criteria for depressive disorders. "OD" signifies once per day. "BD" signifies twice per day.
