# Supplementary material for: Synaptic density marker SV2A is reduced in schizophrenia patients and unaffected by antipsychotics in rats
Source: Nat Commun. 2020 Jan 14;11:246. doi: 10.1038/s41467-019-14122-0 (PMC6959348; doi:10.1038/s41467-019-14122-0)
Supplement: Supplementary file 5 — Reporting Summary [file 41467_2019_14122_MOESM5_ESM.pdf]

nature research

Corresponding author(s): Oliver D Howes

Last updated by author(s): 2019/11/26

Reporting Summary

Nature Research wishes to improve the reproducibility of the work that we publish. This form provides structure for consistency and transparency in reporting. For further information on Nature Research policies, see Authors & Referees and the Editorial Policy Checklist.

Statistics

For all statistical analyses, confirm that the following items are present in the figure legend, table legend, main text, or Methods section.

n/a	Confirmed	
☐	☒	The exact sample size (*n*) for each experimental group/condition, given as a discrete number and unit of measurement
☐	☒	A statement on whether measurements were taken from distinct samples or whether the same sample was measured repeatedly
☐	☒	The statistical test(s) used AND whether they are one- or two-sided<br>*Only common tests should be described solely by name; describe more complex techniques in the Methods section.*
☐	☒	A description of all covariates tested
☐	☒	A description of any assumptions or corrections, such as tests of normality and adjustment for multiple comparisons
☐	☒	A full description of the statistical parameters including central tendency (e.g. means) or other basic estimates (e.g. regression coefficient) AND variation (e.g. standard deviation) or associated estimates of uncertainty (e.g. confidence intervals)
☐	☒	For null hypothesis testing, the test statistic (e.g. *F*, *t*, *r*) with confidence intervals, effect sizes, degrees of freedom and *P* value noted<br>*Give P values as exact values whenever suitable.*
☒	☐	For Bayesian analysis, information on the choice of priors and Markov chain Monte Carlo settings
☒	☐	For hierarchical and complex designs, identification of the appropriate level for tests and full reporting of outcomes
☐	☒	Estimates of effect sizes (e.g. Cohen's *d*, Pearson's *r*), indicating how they were calculated

Our web collection on statistics for biologists contains articles on many of the points above.

Software and code

Policy information about availability of computer code

Data collection	For structural MRI, Siemens Magnetom Prisma software: VE11C; Siemens Siemens 3T Trio software: VB17<br>For PET data: VB40B, Siemens Biograph 6 HiRez<br>Western blot: Image LabTM (PC Version 6.0 SOFT-LIT-170-9690-ILSPC)<br>Confocal imaging: NIS-Elements
Data analysis	PET and MRI: MIAKAT software version 4.3.7<br>Western blot: Image Studio (Version 5.2.5, Li-Cor Biosciences)<br>Autoradiography: MCID Basic 7.0 (Interfocus, Cambridge, UK)<br>Confocal Imaging: ImageJ<br><br>Statistical analysis:<br>GraphPad Prism, version 8.<br>IBM SPSS Statistics, Version 25<br>RStudio Version 1.1.456

For manuscripts utilizing custom algorithms or software that are central to the research but not yet described in published literature, software must be made available to editors/reviewers. We strongly encourage code deposition in a community repository (e.g. GitHub). See the Nature Research guidelines for submitting code & software for further information.

Data

Policy information about availability of data

All manuscripts must include a data availability statement. This statement should provide the following information, where applicable:

- Accession codes, unique identifiers, or web links for publicly available datasets
- A list of figures that have associated raw data
- A description of any restrictions on data availability

The imaging and related clinical data have been deposited on the NODE PET data repository (listed as SV2A imaging in schizophrenia) and are available at: https://molecular-neuroimaging.com/ neuroimaging-database/.

Field-specific reporting

Please select the one below that is the best fit for your research. If you are not sure, read the appropriate sections before making your selection.

☒ Life sciences ☐ Behavioural & social sciences ☐ Ecological, evolutionary & environmental sciences

For a reference copy of the document with all sections, see nature.com/documents/nr-reporting-summary-flat.pdf

Life sciences study design

All studies must disclose on these points even when the disclosure is negative.

Sample size	For human subjects, n = 18 per group (healthy volunteer and schizophrenia)<br><br>For the animal study we treated 3 independent cohorts of animals; cohort 1 and 2 were designed to have a sample size of n=12 per treatment group, and cohort 3 was designed to have n=6 for the control group and n=14 for the Olanzapine group. As some animals had to be culled during the treatment as directed by the veterinary surgeon due to wounds or seromas, the final numbers were:<br>Cohort 1: n=10 for control, n=12 for 0.5mg/kg/day HAL, n=11 for 2.0 mg/kg/day HAL<br>Cohort 2: n=11 control, n=11 for 0.5 mg/kg/day HAL<br>Cohort 3: n=4 control, n=13 for 7.5 mg/kg/day OLZ.<br>For the autoradiography study, only a subset of animals were included due to shortage of the tracer. For this experiment, numbers are: n=7 control, n=3 for 0.5mg/kg/day HAL, n=5 for 2.0 mg/kg/day.<br><br>In both the human and animal experiments, there are no prior data upon which to base a power calculation, given that these are the first experiments comparing SV2A in the living human brain in healthy volunteers and patients with schizophrenia, and exploring the effects of antipsychotic drugs on SV2A in the rat brain. As such, power calculations were not performed.
Data exclusions	No data were excluded from the experiments involving human subjects.<br><br>All data was tested for outliers (Grubbs test, criteria alpha=0.01), but no data fell outside the criteria.<br>In the analysis of immunostaining intensity by confocal imaging, data from one animal in the Olanzapine group of cohort 3 was excluded from the dataset due to poor quality of the staining, resulting in a final sample size of n=4 for control and n=12 for the Olanzapine group.
Replication	Not relevant for clinical arm<br><br>Replication was designed to be determined by the sample size within one cohort: all animals within each cohort were treated at the same time, and the sample size allows for replicate samples within each treatment group. Analysis of various SV2A measures (western blot, 3H-UCB-J tracer binding, and immunostaining intensity) was done on each sample in an identical way to create replicate samples within one group.
Randomization	Not relevant for clinical arm<br><br>On surgery days, animals were randomly assigned to a treatment group. Brains were sectioned and treated for further analysis in random order. Where possible, sample processing was performed while blinded for the treatment group. Where not all samples could be processed in the same day, an equal number of each treatment group was randomly selected for processing in batches.
Blinding	Not relevant for clinical arm<br><br>Researchers were blinded during data acquisition and analysis and where possible during sample processing.

Reporting for specific materials, systems and methods

We require information from authors about some types of materials, experimental systems and methods used in many studies. Here, indicate whether each material, system or method listed is relevant to your study. If you are not sure if a list item applies to your research, read the appropriate section before selecting a response.

Materials & experimental systems

n/a	Involved in the study	
☐	☒	Antibodies
☒	☐	Eukaryotic cell lines
☒	☐	Palaeontology
☐	☒	Animals and other organisms
☐	☒	Human research participants
☐	☒	Clinical data

Methods

n/a	Involved in the study	
☒	☐	ChIP-seq
☒	☐	Flow cytometry
☐	☒	MRI-based neuroimaging

Antibodies

Antibodies used

Western blotting:
Primary antibodies: Rabbit anti-SV2A, Abcam ab32942 (RRID:AB_778192); Mouse anti-GAPDH, Protein tech 60004-1, clone 1E6D9 (RRID:AB_2107436)
Secondary antibodies: Goat anti-Mouse, HRP conjugated, Pierce 31430 (RRID:AB_228307); Goat anti-rabbit, HRP conjugated, Pierce 31460 (RRID:AB_228341).

Immunostaining:
Primary antibody: Rabbit anti-SV2A, Abcam ab32942 (same as for WB).
Secondary antibody: Goat anti-rabbit AlexaFluor555, Abcam ab150090 (no RRID known).

Validation

Mouse anti-GAPDH validated for WB, IC, IHC, ICC, ELISA.
Positive WB detected in: HeLa cells, HepG2 cells, zebrafish tissue, Raji cells, HEK-293 cells, whole Nematode tissue, soybean whole plant tissue, arabidopsis whole plant tissue, whole yeast cells.
Website: https://www.ptglab.com/products/GAPDH-Antibody-60004-1-Ig.htm#validation

Rabbit anti-SV2A validated in: IHC, ICC, WB, IF, IP.
Tested in Mouse, Rat, Human.
Website: https://www.abcam.com/sv2a-antibody-ab32942.html
Specificity of this antibody has previously been verified for immunostaining using SV2A KO mouse brain section, as well as with SV2A blocking peptides (Crevecoeur et al., BMC Neuroscience 2013).

Animals and other organisms

Policy information about studies involving animals; ARRIVE guidelines recommended for reporting animal research

Laboratory animals

Male Sprague Dawley rats, age 6-10 weeks at onset of the treatment (duration of 4 weeks).

Wild animals

The study did not involve wild animals

Field-collected samples

The study did not involve samples collected from the field

Ethics oversight

Animal experiments were carried out in accordance with the Home Office Animals (Scientific Procedures) Act (1986) and European Union (EU) Directive 2010/63/EU, with the approval of the local Animal Welfare and Ethical Review Body (AWERB) panel at King's College London (KCL).

Note that full information on the approval of the study protocol must also be provided in the manuscript.

Human research participants

Policy information about studies involving human research participants

Population characteristics

Thirty-six volunteers (n=18 with schizophrenia [SCZ, 15 male and 3 female] and 18 healthy volunteers [HV, 15 male and 3 female]) completed the study. The groups were not significantly different in age (mean [standard error of the mean, SEM] years SCZ group = 41.5 [2.7], HV group = 38.7 [3.1], p = 0.49). All volunteers with schizophrenia were on antipsychotic medication (mean [SEM] chlorpromazine-equivalent dose = 467.8 [99.3] mg/day, Supplementary Table 1). None of the volunteers with schizophrenia had co-morbid DSM-5 psychiatric diagnoses. The healthy volunteers were screened to exclude any family history of psychosis. Exclusion criteria for all volunteers were: history of neurological disorder, head trauma resulting in a loss of consciousness, drug or alcohol dependence (except for nicotine dependence); significant medical disorder; taking a drug known to interact with SV2A (including levetiracetam, brivaracetam, loratadine or quinine); or contraindications to imaging.

Recruitment

We recruited 18 individuals with a DSM-5 diagnosis of schizophrenia from community mental health services in London. 18 healthy volunteers were recruited through public advertisement.

Ethics oversight

London-West London & GTAC Research Ethics Committee

Note that full information on the approval of the study protocol must also be provided in the manuscript.

Clinical data

Policy information about clinical studies

All manuscripts should comply with the ICMJE guidelines for publication of clinical research and a completed CONSORT checklist must be included with all submissions.

Clinical trial registration	IRAS reference number: 209761; REC reference: 16/LO/1941
Study protocol	Accessible by contacting researchers
Data collection	Recruitment and clinical assessment of healthy volunteers at King's College London – Institute of Psychiatry, Psychology and Neuroscience.<br>Recruitment of patients at South London and Maudsley, Central and North West London, and East London NHS Foundation Trust sites.<br>MRI scanning sessions carried out at the Robert Steiner MRI Unit at Hammersmith Hospital.<br>PET scans carried out at IMANOVA.<br><br>Recruitment and scanning undertaken between 2017 and 2019
Outcomes	[11C]UCB-J volumes of distribution (VT) measured in the frontal cortex, anterior cingulate cortex and hippocampus were the main outcome measures in human subjects, based on the meta-analysis of post-mortem studies showing lower presynaptic protein and mRNA markers in those regions in schizophrenia.<br><br>A two-way analysis of variance (ANOVA) was used to test the effects of group and ROI, and group-by-ROI interactions, on [11C]UCB-J VT and to explore if differences in corrected grey matter volume contributed to our VT findings. Where there were significant effects, planned post hoc independent samples t-tests (two-tailed) were used to test the effect of group on VT and corrected grey matter volume (GMV) at each ROI. For post hoc analyses, a false discovery rate (FDR) (Q) of 5% was used to limit false discoveries when performing multiple comparisons between groups in the three ROIs and FDR-adjusted p values are reported.<br><br>Group differences in clinico-demographic variables were assessed using independent sample t-tests (two-tailed) for normally distributed data and Kolmogorov-Smirnov tests for nonparametric data. We tested if there were significant associations between grey matter VT levels and GMV, symptom severity (PANSS scores), antipsychotic dose and duration of illness using the Pearson product-moment correlation coefficient for normally distributed data, and Spearman's rank correlation for non-normally distributed data. The relationship with antipsychotic dose was explored using chlorpromazine-equivalents derived using the defined daily doses. We conducted exploratory analyses to explore the potential influence of smoking, concomitant medication and clozapine treatment on VT.<br><br>We conducted an exploratory analysis of other brain regions to assess whether there were significant alterations in [11C]UCB-J VT in other brain regions in schizophrenia. We also conducted exploratory analyses of regional distribution volume ratio (DVR), obtained by use of the centrum semiovale as a pseudoreference region. These exploratory analyses were conducted using two-way ANOVA with planned post hoc independent samples t-tests (two-tailed) with FDR correction for multiple comparisons.

Magnetic resonance imaging

Experimental design

Design type	Stuctural T1-weighted MRI
Design specifications	Single T1-weighted structural MRI per subject
Behavioral performance measures	None

Acquisition

Imaging type(s)	Structural
Field strength	3 Tesla
Sequence & imaging parameters	For all patients and 16 of the healthy volunteers, T1-weighted three-dimension magnetisation-prepared rapid acquisition gradient echo (MPRAGE) images were acquired on a Siemens Magnetom Prisma 3T scanner (Siemens, Erlangen, Germany) according to the following parameters: repetition time = 2300.0 ms, echo time = 2.28 ms, flip angle = 9 °, field of view (FOV) = 256 × 256 mm, 176 sagittal slices of 1 mm thickness, distance factor = 50%, voxel size = 1.0 ⍰ 1.0 ⍰ 1.0 mm. For 2 healthy volunteers, T1-weighted three-dimension MPRAGE images were acquired on a Siemens 3T Trio clinical MRI scanner (Siemens Healthineers, Erlangen, Germany) with the following parameters: repetition time = 2300.0 ms, echo time = 2.98 ms, flip angle = 9 °, FOV = 256 × 256 mm256, 160 sagittal slices of 1 mm thickness, distance factor = 50%, voxel size = 1.0 ⍰ 1.0 ⍰ 1.0 mm.
Area of acquisition	Whole brain scan

Diffusion MRI ☐ Used ☒ Not used

Preprocessing

Preprocessing software

MIAKAT software version 4.3.7 (http://www.miakat.org/MIAKAT2/index.html), which implements MATLAB (version R2016a; Mathworks Inc., Natick, MA, USA), FSL (version 5.0.10; FMRIB, Oxford, UK) functions for brain extraction and Statistical and Parametric Mapping12 (Wellcome Trust Centre for Neuroimaging, http://www.fil.ion.ucl.ac.uk/spm) functions for image segmentation and registration.

Normalization

The aim of MRI pre-processing in this study is to obtain a set of regions of interest in subject space. In order to achieve this a template MRI image is nonlinearly warped from MNI152 space to each subjects' MRI (which has been rigid-body registered to MNI space). The parameters from this nonlinear warping are applied to the CIC atlas which is also in MNI152 space. Therefore the only normalisation that was applied to the MRI images was a linear rigid-body registration to MNI space.

We use this methodology because the aim in this study is to manipulate the PET imaging data as little as possible as this is what is used for quantification of SV2A (our primary interest). The rigid-body registration is applied so that orientation of the brains is consistent for visualisation purposes. It is important to note that rigid-body registration does not tamper with the intensities of the PET data, which is the primary focus here.

Normalization template

ICBM152

Noise and artifact removal

None

Volume censoring

None

Statistical modeling & inference

Model type and settings

In our second level analyses, we used two-way ANOVA to test the effects of group (schizophrenia and healthy volunteer) and ROI, (frontal cortex, anterior cingulate cortex and hippocampus) and group-by-ROI interactions, on [11C]UCB-J VT and to explore if differences in corrected grey matter volume contributed to our VT findings. Where there were significant effects, planned post hoc independent samples t-tests (two-tailed) were used to test the effect of group on VT and corrected grey matter volume (GMV) at each ROI. For post hoc analyses, a false discovery rate (FDR) (Q) of 5% was used to limit false discoveries when performing multiple comparisons between groups in the three ROIs. We tested if there were significant associations between grey matter SV2A levels and GMV using the Pearson product-moment correlation coefficient for normally distributed data, and Spearman's rank correlation for non-normally distributed data.

We conducted an exploratory analysis of other brain regions (dorsolateral prefrontal cortex, temporal, parietal and occipital lobes, thalamus and amygdala) to assess whether there were significant alterations in [11C]UCB-J VT in other brain regions in schizophrenia, and an exploratory analysis of regional distribution volume ratio (DVR), using two-way ANOVA with planned post hoc independent samples t-tests (two-tailed) with FDR correction for multiple comparisons.

Effect(s) tested

Effects of group (schizophrenia and healthy volunteer) and ROI, (frontal cortex, anterior cingulate cortex and hippocampus) and group-by-ROI interactions, on [11C]UCB-J VT and corrected grey matter volume
Effect of group on VT and corrected grey matter volume (GMV) at each ROI.

Associations between grey matter VT levels and GMV symptom severity (PANSS scores), antipsychotic dose and duration of illness.

Effects of smoking, concomitant psychotropic medications and clozapine use on VT.

Exploratory analysis for effects of group, ROI and group-by-ROI interactions [11C]UCB-J VT in other brain regions (dorsolateral prefrontal cortex, temporal, parietal and occipital lobes, thalamus and amygdala).

Group comparison of VT in the centrum semiovale (pseudoreference region).

Exploratory analysis for effects of group, ROI and group-by-ROI interactions [11C]UCB-J DVR in main (frontal and anterior cingulate cortices and hippocampus)a nd secondary (dorsolateral prefrontal cortex, temporal, parietal and occipital lobes, thalamus and amygdala) ROIs.

Specify type of analysis: ☐ Whole brain ☒ ROI-based ☐ Both

Anatomical location(s)

Automated labelling used to explore the frontal cortex, anterior cingulate cortex, hippocampus, dorsolateral prefrontal cortex, temporal, parietal and occipital lobes, thalamus, amygdala and centrum semiovale

Statistic type for inference
(See Eklund et al. 2016)

Not applicable

Correction

FDR

Models & analysis

n/a	Involved in the study
☒	☐ Functional and/or effective connectivity
☒	☐ Graph analysis
☒	☐ Multivariate modeling or predictive analysis
